# Diagnostic accuracy and acceptability of molecular diagnosis of COVID-19 on saliva samples relative to nasopharyngeal swabs in tropical hospital and extra-hospital contexts: The COVISAL study

**DOI:** 10.1371/journal.pone.0257169

**Published:** 2021-09-13

**Authors:** Mathieu Nacher, Mayka Mergeay-Fabre, Denis Blanchet, Orelie Benois, Tristan Pozl, Pauline Mesphoule, Vincent Sainte-Rose, Véronique Vialette, Bruno Toulet, Aurélie Moua, Mona Saout, Stéphane Simon, Manon Guidarelli, Muriel Galindo, Barbara Biche, William Faurous, Laurie Chaizemartin, Aniza Fahrasmane, Devi Rochemont, Fode Diop, Moussa Niang, Jean Pujo, Nicolas Vignier, Dominique Dotou, Astrid Vabret, Magalie Demar

**Affiliations:** 1 CIC INSERM 1424, Centre Hospitalier de Cayenne Andrée Rosemon, Cayenne, French Guiana; 2 DFR Santé, Université de Guyane, Cayenne, French Guiana; 3 Laboratoire, Centre Hospitalier de Cayenne Andrée Rosemon, Cayenne, French Guiana; 4 Centre de Ressources Biologiques (CRB) Amazonie, Centre Hospitalier de Cayenne Andrée Rosemon, Cayenne, French Guiana; 5 Unité mixte de recherche TBIP, Université de Guyane, Cayenne, French Guiana; 6 Centre délocalisé de prévention et soins de Maripasoula, Maripasoula, French Guiana; 7 Service des Urgences, Centre Hospitalier de Cayenne Andrée Rosemon, Cayenne, French Guiana; 8 Service de Gynécologie Obstétrique, Centre Hospitalier de Cayenne Andrée Rosemon, Cayenne, French Guiana; 9 Service de Virologie, CHU de Caen, Caen, France; University of Helsinki: Helsingin Yliopisto, FINLAND

## Abstract

A prospective study was conducted among different intra and extra-hospital populations of French Guiana to evaluate the performance of saliva testing compared to nasopharyngeal swabs. Persons aged 3 years and older with mild symptoms suggestive of COVID-19 and asymptomatic persons with a testing indication were prospectively enrolled. Nasopharyngeal and salivary samples were stored at 4°C before analysis. Both samples were analyzed with the same Real-time PCR amplification of E gene, N gene, and RdRp gene. Between July 22th and October 28th, 1159 persons were included, of which 1028 were analyzed. When only considering as positives those with 2 target genes with Ct values <35, the sensitivity of RT-PCR on saliva samples was 100% relative to nasopharyngeal samples. Specificity positive and negative predictive values were above 90%. Across a variety of cultures and socioeconomic conditions, saliva tests were generally much preferred to nasopharyngeal tests and persons seemed largely confident that they could self-sample. For positive patients defined as those with the amplification of 2 specific target genes with Ct values below 35, the sensitivity and specificity of RT-PCR on saliva samples was similar to nasopharyngeal samples despite the broad range of challenging circumstances in a tropical environment.

## Introduction

French Guiana is a sparsely populated French Overseas territory between the state of Amapa in Brazil and Suriname. It has a rapidly growing population nearing 300 000 persons occupying a territory with a similar size as England [1]. Its Health System is well funded but it has a limited hospital capacity that could be overwhelmed by COVID-19 epidemic surges. Moreover, over half of the population lives below the poverty line, and 20% live surrounded by the Amazon Forest in remote villages of the interior of French Guiana. Furthermore, the mosaic of cultures in French Guiana encapsulates diverse representations of health and disease [2], that may also impact adherence to public health authorities’ recommendations. In June of 2020, the epidemic surge in Brazil eventually reached this small territory and threatened to overwhelm its limited health infrastructure and human resources [3]. Furthermore, diagnostic capacity was also strained by a simultaneous dengue epidemic that increased patient waiting time for diagnosis [4]. In July 2020, as the epidemic peaked in French Guiana, health authorities struggled to expand hospital and Intensive Care Unit capacity, to continue contact tracing and quarantine patients that were unable to isolate themselves at home in hotels, and to reduce testing bottlenecks at the public and private laboratories on the territory to expand COVID-19 testing. The implementation of tailored curfews and the predominantly young population allowed to avoid overwhelming the health system and led to a relatively low case-fatality rate in comparison with other Amazonian territories [3]. The repeated epidemic waves, air-transportation requirement for COVID-19-testing strained the local testing structures of French Guiana. Diagnosis, contact-tracing and patient isolation have required massive testing efforts and ways to optimize processes have been a permanent concern for health authorities, which are still bracing for the next wave coming either from Brazil or from France.

Reverse-transcriptase polymerase chain reaction (RT-PCR) from a nasopharyngeal swab specimen remains the main diagnostic method for COVID-19 [5]. However, the collection of a nasopharyngeal swab is labor and equipment-intensive, slowing down the collection of samples. In addition, it is an unpleasant procedure, associated with waiting delays for swab collection–often in crowded stressful places—which may discourage some persons to get tested or to repeat tests, if necessary. New testing strategies to rapidly identify cases are urgently needed to reduce waiting delays, and facilitate mass screening. The collection of saliva samples is easy and painless, it does not require trained staff and may allow self-sampling. To reveal the presence of SARS-COV-2 in saliva, some have used antigen rapid tests [6] whereas others relied on the amplification of genetic material from different types of samples [7]. The review of studies comparing real time PCR results on salivary and nasopharyngeal samples yields variable results, often showing greater sensitivity and lower RT-PCR Cts in nasopharyngeal swab samples [8–10] but sometimes showing on the contrary greater sensitivity in saliva samples [11, 12]. Recently a meta analysis comparing nucleic acid amplification between nasopharyngeal swabs and saliva samples using Bayesian latent class analysis estimated that there was little difference in sensitivity between nasopharyngeal swabs (pooled sensitivity = 85.7% (95% credible interval, 76.5%-93.4%)) and saliva (pooled sensitivity = 85.6%, 95% credible interval, 77%-92.7%). Among the subgroup of ambulatory patients pooled sensitivity was 84.5% for saliva versus 88% for nasopharyngeal samples [7]. There are many potential sources of variation that can explain discrepancies between individual studies: differences in study population (hospitalized patients versus screening of contacts or mildly symptomatic patients), differences in nasopharyngeal or saliva collection techniques and timing, differences in conditioning and delays in processing raw saliva samples, or differences in the RT-PCR techniques used.

Shortly after the peak of the epidemic in French Guiana in July 2020, a prospective study was conducted to evaluate the performance of saliva testing compared to nasopharyngeal swabs. In the context of a public health emergency, a first analysis was performed to look at sensitivity of saliva samples compared to nasopharyngeal samples and contribute to the Haute Autorité de Santé’s and the Ministry’s decisions regarding these tests [13, 14].

The study continued until it reached the desired sample size in order to refine estimations of diagnostic accuracy and to analyze the respective acceptability of nasopharyngeal and saliva sampling in contrasted screening contexts in French Guiana.

## Methods

### Context in French Guiana

At the time of the study, 3.2% of the population of French Guiana had had a confirmed COVID-19 infection, notably the poorest populations [15]. In this epidemiologic context, testing and tracking were implemented throughout the epidemic, mobile teams including the remote health centers, the Red Cross, Médecins du Monde, and the reinforcements from the Réserve Sanitaire were coordinated by the regional health agency to perform COVID-19 testing and investigate around clusters of cases. The testing efforts hence peaked to nearly 0.5% of the population screened in a day [3].

### Study conduct

Between July 22^th^ and October 28^th^, persons aged 3 years and older with mild symptoms suggestive of COVID-19 and asymptomatic persons with a testing indication were prospectively enrolled, at various testing sites and mobile testing brigades in French Guiana. The furthest site was a township located 240 km from Cayenne in the Amazonian Forest only accessible by canoe or plane. The mobile testing teams, consisting of healthcare personnel (doctors, nurses) were coordinated by the Health Regional Agency, targeted villages and neighborhoods with active transmission, often testing persons often out of doors or in health centers. These mobile teams included staff from the Red Cross, Médecins du Monde, the Cayenne hospital PASS, the Maripasoula health center, and—reinforcements from mainland France—the health reserve. Team projection was organized each week by the Health Regional Agency of French Guiana according to the latest knowledge of current clusters of cases. Screening teams were hence sent to the concerned neighborhoods—urban or rural, and usually socially disadvantaged; furthermore, patients requiring hospitalization for other reasons than obvious COVID-19 (for example fractures or pregnancies) were screened to rule out infectiousness; drive through testing services were also deployed to offer testing to any person requesting a test during the epidemic peak.

### Inclusion and exclusion criteria

The inclusion criteria were: males or females aged at least 3 years old with an indication for a COVID-19 diagnostic test (contact case, systematic screening, symptoms, etc.). The non-inclusion criteria were: taking treatments that reduce salivary volume (anticholinergic activity), impossibility of performing the Nasopharyngeal swab, being under guardianship or curatorship, or placed under protective measures, and patient (or his/her legal representative) refusal to participate.

#### Patient enrolment

Study participants were then enrolled and sampled in accordance with the protocol. The investigators–physicians, midwives, and residents or nurses under medical supervision—explained the study, its objectives, and obtained the oral consent of the patient or his/her legal representative, as required by the Ethical committee. The form was completed by the investigator or delegated to paramedical staff by the investigator. The patients were advised to accumulate saliva in their mouth before spitting it in the dedicated container. Trained nurses performed the nasopharyngeal swab–which were placed in transport medium—and collected the salivary sample in a urine container without any particular transport medium. The saliva sample volume, its appearance, the requirement for dilution, the time at collection, the time of arrival at the hospital, the time of analysis, and when the samples were frozen were registered. Paired saliva and nasopharyngeal samples were biobanked for verification with other methods in Caen University Hospital, and to serve as a resource to evaluate the diagnostic accuracy of future tests.

A trained agent carried out a short questionnaire exploring the acceptability of both sampling methods, and the willingness to repeat at test or not, and reasons for doing so, age, sex, notion of symptoms of contacts, medical history putting the patient at risk of severe infection, notion of drinking or eating before the test, and mouth rinsing. Research samples and participant information did not allow patient identification and were collected with a unique identifying number and entered in an anonymized database (ENNOV system in compliance with the Food and Drugs Administration 21CFR norm). Independently from research, results from the nasopharyngeal sample were transmitted to the field to give them back to the patient, and act-upon if necessary.

All completed forms and samples were sent to Cayenne hospital at the end of each day without any particular transport medium and samples were stored at 4°C before analysis. Because of the different contexts in which the persons were tested the analysis took place at different time intervals after sampling, depending notably on transport constraints or staff reductions on weekends.

### Laboratory analysis

Both samples were analyzed with the same Real-time PCR assay throughout the study using the QIAsymphony and GeneFinder kit. The commercial GeneFinder COVID-19 Plus RealAmp kit (ELITechGroup, Puteaux, France) amplifies and permits the SARS-CoV-2 RNA detection especially the viral RdRp, E and N genes as well as the Human housekeeping gene RNAse P as internal control. Cut-offs for positivity were pre-specified by the manufacturers of the commercial kits. The kit showed a high sensitivity of 97.4% (84.6 to 99.9%; 95% CI), with a limit of detection (LOD) of 10 copies per 25 μL reaction, for all the target viral genes and no cross-reactivity, i.e. specificity of 100% (97.6 to 100%; 95% CI) with 20 common human respiratory viruses, including four other human coronaviruses (OC43, 229E, HKU-1 and NL63). The clinical performance was tested using 60 individual upper respiratory specimens and 60 sputum specimens collected from patients with signs and symptoms of a respiratory infection showed a Positive Percent Agreement of 100% (95% CI: 88.6% - 100%) and a Negative Percent Agreement 100% (95% CI: 88.6% - 100).

The viral RNA extractions were automated using the Qiasymphony system (QIAGEN, Hilden, Germany) with a final elution of 50μl. Following manufacturer’s instructions, the RT-PCR used 5 μl of RNA template into 15 microliters of a ready-to-use mix. The PCR program comprised two different steps. Step 1 ran 1 cycle at 50°C for 20 min and 1 cycle at 95°Cfor 5 min. Step 2 presented 45 cycles of 95°C for 15 s and 58°Cfor 60 s. Valid results were defined as amplification of the internal control gene with a Ct≤35. A sample was considered positive if at least one of the RdRp, N or E genes were amplified with a Ct≤40 whereas negative samples were defined as no amplification of any viral genes with an amplified internal control with a Ct≤35 [16]. Viral nucleic acid was extracted by using the Qiagen DSP QIAsymphony DSP Virus/Pathogen Mini Kit and the Cellfree200_V7_DSP QIAsymphony SP Protocol on the QIAsymphony RGQ, an integrated fully automated nucleic acid extraction (chemical lysis and paramagnetic bead binding) and sample preparation platform (Qiagen GmbH, Germany). An Applied 7500 cycler (Thermofisher) was used. Nucleic acid extraction methods could affect the results of viral nucleic acid amplification tests, thus we treated the saliva-nasopharyngeal specimen couples with the same method and, most of the time, in the same series. Discordant results did not lead to repeated analysis. The eluates were obtained from 200μl of specimens (300 μL minus 100 μL of dead volume). There was no inactivation step in the preanalytical processing stage. When saliva specimens presented high viscosity, they were fluidified with proteinase K using a 10% equivalent volume of the specimen, then vortexed and incubated at 56°C during 15 minutes. If the collected volume of saliva was insufficient (< 1ml), we completed up to 1 mL with NaCl 0,9%. All these processes including the total collected volume of saliva, the saliva consistence (fluid or viscous) were registered in order to determine the impact of these pre-analytic conditions.

The remainder of each sample was then divided into paired aliquots and biobanked for further studies of new diagnostic tools.

### Statistical analysis

Statistical analysis was performed using STATA® 16 (Stata corporation, College Station, Texas, USA). Cross tabulations considering different subgroups were performed. The gold standard was the result on the nasopharyngeal test. Sensitivity, specificity, positive and negative predictive values were computed. We considered the RdRp and N genes–which are specific for SARS-Cov-2—to calculate different Ct categories. Cohen’s kappa was computed for different definitions of “positive”. Discordant results were analyzed by crosstabulations or t-tests in order to identify potential variables of interest in explaining differences. The responses to the questionnaires were analyzed as simple frequencies and percentages but were also cross-tabulated with variables that might affect preferences. The relation between age and saliva volume was measured using Spearmans’ correlation. The Sign rank test was used to compare target gene Cts in nasopharyngeal and saliva samples.

### Ethical

The protocol received ethical approval from the Comité de Protection des Personnes Sud Méditerranée II under the number 2020-A02009-30/SI:20.07.07.54744. It was classified as a research involving human persons of the third category and complied with the “methodologie de reference” MR003 from the Commission Nationale Informatique et Libertés.

## Results

### General results

#### Study period and sites

**Fig 1:**
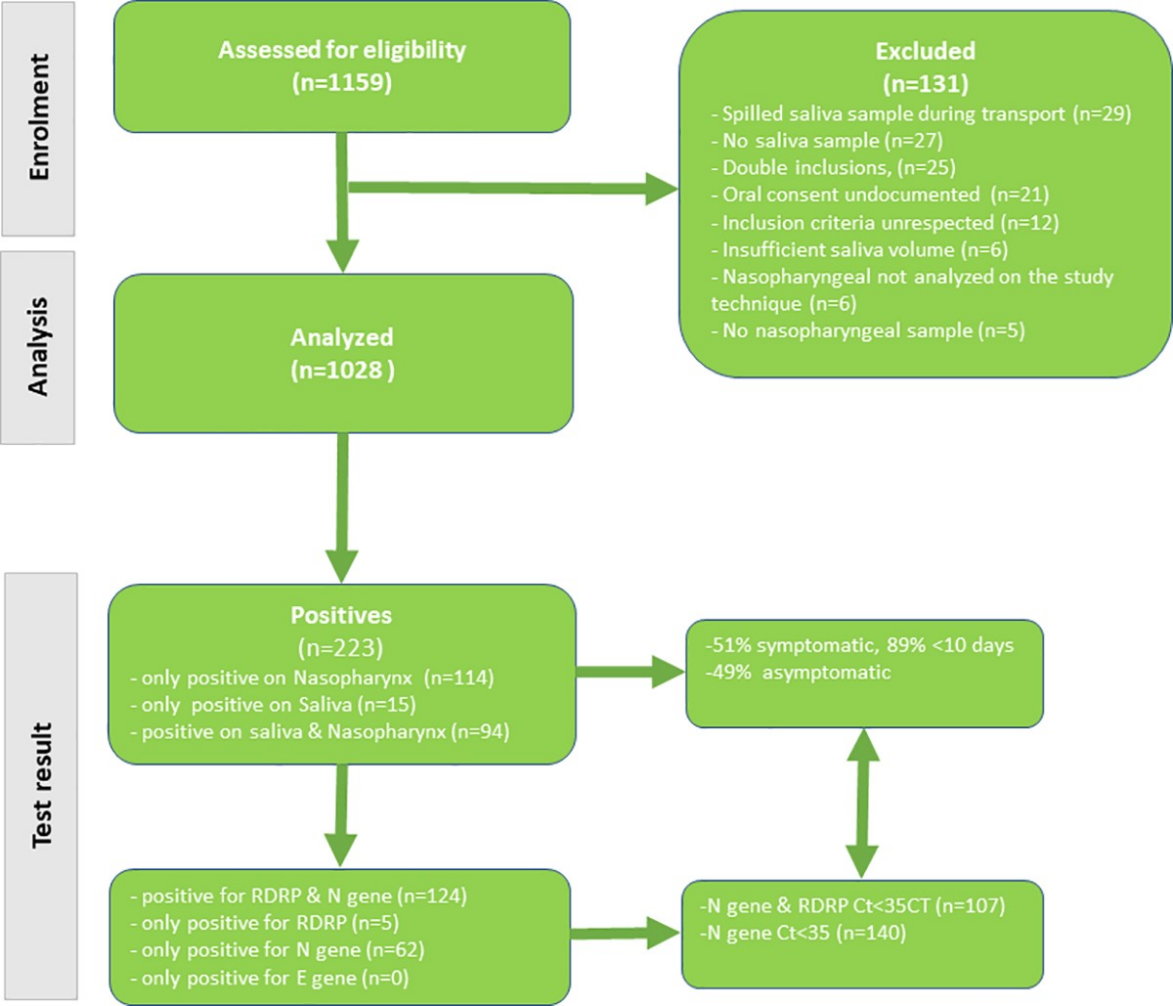
COVISAL study flowchart. The top half of the flowchart shows the cascade of the number of patients screened and the number of patients that were included in the analysis and the reasons why some were excluded. The bottom half of the flowchart shows the number of ‘positives’ (according to the manufacturer) by type of sample and the proportion of symptomatic patients and among these those that had symptoms less than 10 days before inclusion. Finally, the bottom left of the flowchart breaks down positives by target gene and the bottom right shows the number of positives with Ct <35, which are assumed to be most infectious and most important to detect.

Between July 22^th^ and October 28^th^, 1159 persons were included in this research, of whom 1028–575 females (56%) and 453 males (44%)—were analyzed. (Fig 1).

Of these, 108 (10.5%) were sampled at Cayenne hospital (outside of obstetrics), 95 (9.2%) were sampled at the obstetrical ward, 14(1.4%) were sampled by the Red Cross mobile team, 406 (39.5%) were sampled by Doctors of the World mobile teams (Médecins du Monde), and 405(39.4%) were recruited by the team in Maripasoula remote village at the peak of transmission, 240 km from Cayenne.

There were 17 (7.6%) positive tests in Cayenne hospital, 7 (3.1%) at the obstetrical ward, 1(0.5%) at the Red Cross, 46 (20.6%) at Doctors of the World, and 152(68.2%) at Maripasoula.

#### Patient characteristics

The median age was 34 years (Interquartile range = 23–47, minimum 3 years-maximum 88 years). The motive for doing a test was symptoms for 345 persons (33.5%); there were 277 contact cases (26.9%), of whom 140 (50.5%) were symptomatic. Among the reported conditions, there were 119 (11.5%) with known hypertension, 58(5.6%) diabetic patients, 41 were obese (4%), 10 (1%) immunocompromised persons, and 111(10.8%) pregnant women.

#### Sample volume and transport delays

**Fig 2:**
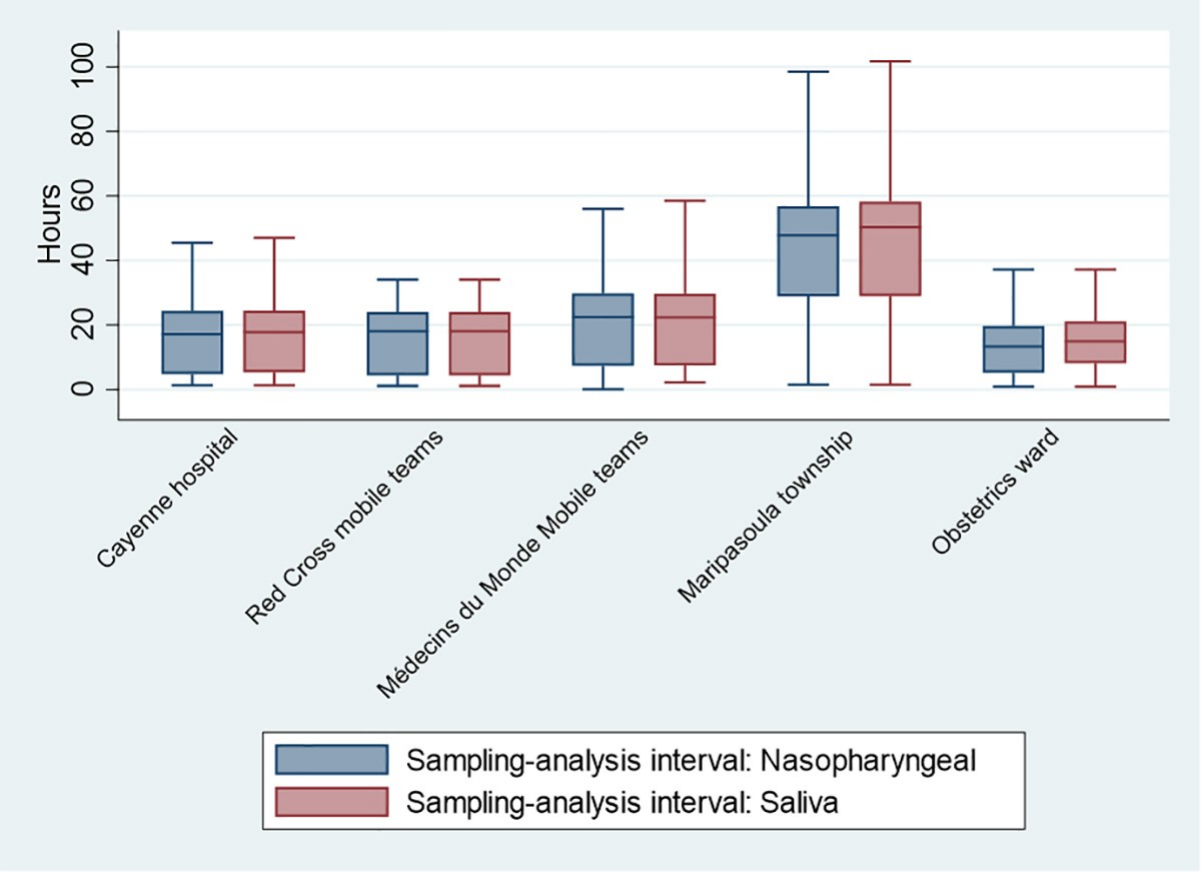
Interval between sample collection and analysis.

There was no significant relation between age and salivary sample volume (Spearman’s Rho = -0.04, P = 0.13). Overall, 192 samples (18.7%) were diluted with normal saline (mean volume = 504 ml ±234 ml). It was logical that samples from Maripasoula–which lies 240 km in the forest and requires air transport to transfer samples—were processed with significantly longer delays (Fig 2), but among positives for at least one sample type, there was no significant (P = 0.9) difference in discordance between nasopharyngeal and saliva samples.

*‘Positives’*. Overall, there were 223 ‘positive’ (Ct< = 40) results; among these, 208 (93.3%) were from nasopharyngeal samples and 109 (48.9%) from saliva samples. (Fig 1, Table 1) Of these 223 ‘positives’, only 1 of 3 gene target was amplified for 67 patients (30%) (62 N gene, 5 RdRp); among ‘positives’ 113 (50.7%) had symptoms for over 10 days before the test, and 83 (37.5%) had Ct values over 35 –a threshold beyond which patients have been deemed no longer contagious [17].

**Table 1:** RT-PCR positive results for nasopharyngeal and saliva samples for different definitions of positivity.

*Positive definition*	Nasopharyngeal positive	Nasopharyngeal negative
**Any gene with Ct< = 40, any delay, any presentation**		
**Saliva positive**	94	15
**Saliva negative**	114	805
**Any gene with Ct< = 40 & Symptoms <10 days**		
**Saliva positive**	64	6
**Saliva negative**	24	187
**At least 2 target genes Ct < = 40 & Symptoms <10 days**		
**Saliva positive**	57	6
**Saliva negative**	16	187
**2 genes (RdRp & N gene) with Ct <35, any delay, any presentation**		
**Saliva positive**	83	1
**Saliva negative**	0	944

### Discordant results

Among those with a positive result, discordant results between nasopharyngeal samples and saliva samples were more likely to be observed among those included by Doctors of the World (AOR = 6.6 (95%CI = 1.6–26.7), and those without symptoms (AOR = 9.9 (95%CI = 5.1–19.2). Among positives, the mean delay between sample collection and analysis was surprisingly *shorter* among discordant results (41.5±26.5 hours) than among concordant results (49.2±23.2 hours), P = 0.04. The volume of saliva was not significantly different between discordant (1391 ± 1073 microliters) and non-discordant results (1507 ± 1089 microliters), P = 0.43. Among those with a positive result, there was no significant difference of the frequency of discordant results between samples coming from the hospital or outside of the hospital (P = 0.7). Having had something to drink or to eat within 30 minutes before the test, and rinsing one’s mouth or not, were not significantly associated with increased proportion of discordant results between nasopharyngeal and saliva tests.

### Diagnostic accuracy

The sensitivity–using nasopharyngeal samples as gold standard—went from very poor among asymptomatic patients (for any positive gene, any Ct), to 100% when only considering patients with 2 target genes with Ct values <35 (Table 2). The positive predictive values and agreement were very above 98% (Table 2). Only 29% of patients that were positive and asymptomatic had Ct values for 2 genes<35 whereas 65.8% of symptomatic patients had Ct values for 2 genes<35, P<0.0001. The median Ct for the positive N gene target was 21.5 (range = 12–41) in nasopharyngeal samples versus 26.1 (range = 13–39) in saliva samples, P = 0.003; for the E gene target the median was 21.5 (range = 13–36) in nasopharyngeal samples versus 24.6 (range = 11.4–38.7) in saliva samples, P = 0.02; for the RdRp gene target the median was 22.5 (range = 14.3–41.7) in nasopharyngeal samples versus 26.3 (range = 13–40.9) in saliva samples, P = 0.005.

**Table 2:** Diagnostic accuracy of COVID-19 RT-PCR on saliva samples relative to nasopharyngeal samples considering different patient groups and Ct thresholds.

	Sensitivity (95% CI)	Specificity (95% CI)	Positive predictive value (95% CI)	Negative predictive value (95% CI)	Cohen’s Kappa
Asymptomatic, any gene, any Ct, any delay (N = 90 positives)	16.8 (10.1–25.6)	98.9 (97.7–99.6)	73.9 (51.6–89.8)	87.2 (84.4–89.7)	0.23
Any gene, any Ct any delay (N = 223 positives)	45.4 (38.6–52.5)	98.7 (97–98.9)	86.2 (78.3–92.1)	87.6 (85.3–89.7)	0.52
Symptoms <10 days (N = 94 positives)	72.7 (62.2–81.7)	96.9 (93.3–98.8)	91.4 (82.3–97.8)	88.6 (83.5–92.6)	0.73
Symptoms <10 days & at least 2 positive target genes (N = 79 positives)	78.1 (66.9–86.9)	96.9 (93.3–98.9)	90.5 (89.4–96.4)	92.1 (87.5–95.4)	0.78
2 positive target genes with Ct<35 (N = 84 positives)	100 (95.6–100)	99.9 (99.4–100)	98.8 (93.5–99.9)	100 (99.6–100)	0.98

**Fig 3:**
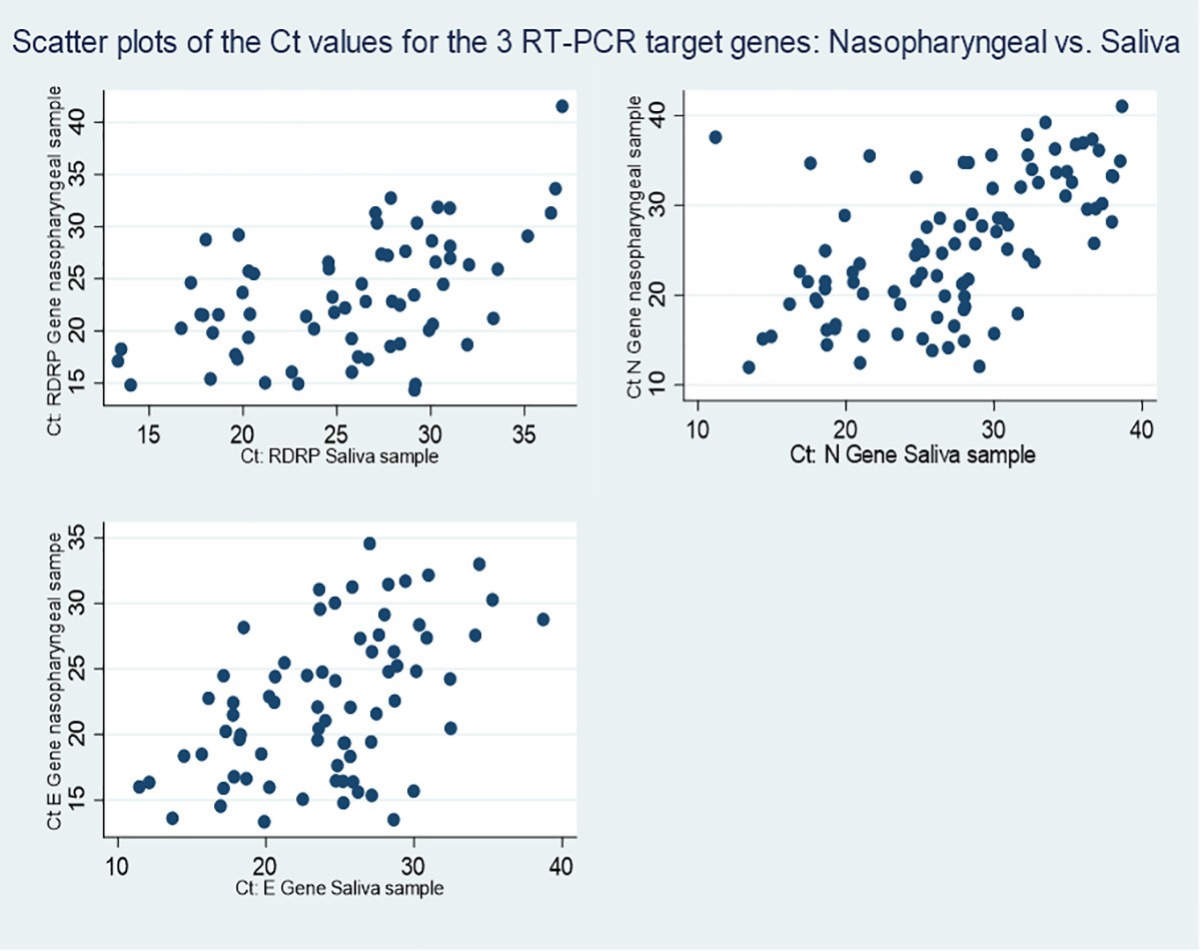
Different gene target Cts on saliva and nasopharyngeal samples. Scatterplots between gene target Ct results for nasopharyngeal (y axis) and saliva samples (x axis) for RdRp Gene (top left), N Gene (top right), and E Gene (bottom left).

Scatterplots of the Cts of the 3 target genes measured in nasopharyngeal and saliva samples show a gradually increasing dispersion for higher Ct values up to the originally accepted threshold of 40 (Fig 3). Spearman’s correlation coefficients between saliva and nasopharyngeal samples of positive patients were +0.58, P<0.0001 for the N gene target, +0.47, P<0.0001 for the E gene target, and +0.45, P = 0.0001 for the RdRp gene target.

### Patient preferences (S1 Appendix)

#### Repeating nasopharyngeal test

Over 63% of patients declared that if their test was negative, they would be willing to repeat a test based on a nasopharyngeal sample but 17.8% would refuse to do so. Women were more likely than men to refuse to do another nasopharyngeal test (20.8% versus 13.9%, p = 0.003). Patients aged over 40 years were less likely to refuse to do another nasopharyngeal swab (10.3%) than those in age groups between 20 and 40 years (21.5%), and those <20 years (23.5%). When adjusting for age, inclusion site using multinomial regression, sex was no longer associated with refusal to do another test; younger age groups remained more likely to refuse and persons included in Maripasoula (ARR = 0.5 (95% CI = 0.3–0.9)) or in precarious neighborhoods of Cayenne by Doctors of the World (ARR = 0.3 (95% CI = 0.16–0.57))) were less likely to refuse than those seen at the hospital. Among those who gave a reason for their refusal 83% declare it was because it was unpleasant and 7.8% because of the waiting time.

#### Test preference

When asked if–under the premise that both samples were of equal performance—nearly 2/3rds (65.6%) of patients preferred the salivary test (68.9% women vs 61.5% men) and 11.1% preferred the nasopharyngeal test (9% women vs 13.7% men). Indeed 76.3% of participants would be willing to repeat a salivary test while only 63% would repeat a nasopharyngeal test. Among those who said they would refuse another nasopharyngeal test, persons aged over 40 years were less likely (68.4%) to cite unpleasantness as a motive for refusal to take another nasopharyngeal test than age groups <20 years (89.7%), and 20–40 years (87.5%).

#### Confidence in ability to perform self-sampling

Among the respondents to the question whether patients would feel capable of taking the sample alone, 6% said they could not, 67% said it would be very easy, and 17.5% felt they would probably be able to do it.

## Discussion

The present results show that, in a diverse set of circumstances the sensitivity of RT-PCR on salivary samples relative to nasopharyngeal samples ranged from very poor to excellent depending on the assumptions of what was a “positive” nasophayngeal RT-PCR result—our gold standard. Similarly, Cohen’s kappa values ranged from fair agreement to almost perfect agreement. Ct values on nasopharyngeal and saliva samples were positively correlated but the Ct values for all target genes were always significantly lower in nasopharyngeal samples than in saliva samples. The method we used amplified 3 gene targets, 2 of which were specific to COVID-19, and it was initially recommended to stay with the manufacturer’s recommendations that positives could have CT values up to 40. However, with accumulating knowledge there have been rapid changes in the interpretation of results [18–20]. When only 1 of 3 genes is positive and Ct values are over 37, the French Society for Microbiology has advised to conclude to a negative result; Ct values between 33 and 37 are considered weak positives. An earlier study suggested a Ct threshold under 35 [17]. Hence, when considering any patient with 2 positive specific target genes with CT values<35, sensitivity was nearly 100%, irrespective of the presence or absence of symptoms–a very different interpretation from the same data [13]. Specificity and epidemic context-specific predictive values were high—generally between 85 and 100% depending on the population selected. Although, at the individual level there may be exceptions [21], Ct levels indirectly correlate with the risk and duration of transmission [17, 22] which makes the good performance of Rt PCR on saliva samples in those with Ct values<35 is important.

Among samples with at least one positive result, delays between sampling and analysis were not associated with discordant results; the factors independently associated with discordant results were: choosing a Ct cutoff of 40, not having any symptoms and samples collected by Doctors of the World. These mobile teams worked in the most precarious areas around Cayenne, often working long hours in the sun, which perhaps heated cooler boxes above 4°C and constituted suboptimal storage conditions before analysis.

These results seem consistent with meta-analyses which observed a difference of sensitivity of RT-PCR generally in favor of nasopharyngeal samples [7, 23, 24]. However, these systematic reviews showed the great heterogeneity of results between studies with many potential differences that were not accounted for. Beyond the sources of variability addressed here–population, type and timing of saliva sample, presence or absence of transport medium, delays, gene targets and equipment used—a major one is hence the actual definition of what constitutes a “positive”, something that was not so well defined a few months ago but has become increasingly consensual as knowledge of the natural history of the infection and the transmission potential are better understood. Overall, when parting from the strict manufacturer recommendations and aligning to current recommendations for interpretation, the difference between RT-PCR results on nasopharyngeal and saliva samples became negligible, suggesting that for potentially contagious patients, saliva samples were as contributive as nasopharyngeal samples. Perhaps future meta-analyses should use data tables taking into account Ct thresholds to compute summary measures.

Despite the apparent robustness of our results across these contrasted settings, the variations in saliva volume, in the timing relative to food or drink, in delays for sample processing may have introduced some variability in the results that would have required a greater number of positives. Systematically repeating sampling and analyses in discordant results could have reduced discordant results but it was not possible given the remoteness of the study sites and the overstretched workforce. Despite these limitations, the results seemed coherent with the literature, which generally showed the very high sensitivity of RT-PCR on saliva samples relative to nasopharyngeal samples [7, 23–25]. Some authors have shown that it was even possible to pool up to 6 saliva samples and retain high sensitivity, a finding that could facilitate mass screening [26].

Questionnaires showed that salivary sampling was much preferred to nasopharyngeal sampling, mostly because it avoided the unpleasantness of the procedure. This led to a lower rate of intended refusals and persons were more likely to repeat a salivary test if necessary. Younger age groups were more likely to refuse and to state that the nasopharyngeal sample collection was unpleasant than persons aged over 40 years—perhaps feeling more at risk of severe complications they were less likely to be dissuaded by the mild discomfort associated with the diagnosis. The premise that persons from different cultures had different representations of various illnesses [2] and were less likely to adhere to “western medicine” seemed to be contradicted by the finding that persons included in the poorest neighborhoods–often immigrants from South America and the Caribbean—and persons from Maripasoula–mostly Amerindian and Maroon populations—were less likely to refuse another test than persons included in the hospital groups. Besides culture, another potential explanation could be that, in these field-testing interventions, the population were more concerned by COVID-19 because it affected their neighborhood and thus more likely to follow health professional’s recommendations. Finally, most persons thought they would not have difficulties to collect the sample on their own. However, these theoretical questions about the sample type did not unfold the practical aspect of giving timely results–especially in the most remote parts where molecular biology facilities are not available; rapid antigen testing, even on nasopharyngeal samples, therefore has the great advantage of giving rapid results.

In conclusion, when defining positive patients as those with the amplification of 2 specific target genes with Ct values below 35, the sensitivity and specificity of RT-PCR on saliva samples was similar to nasopharyngeal samples despite the broad range of challenging circumstances in a tropical environment and independently of the presence of symptoms. Across a variety of cultures and socioeconomic conditions, saliva tests were generally much preferred to nasopharyngeal tests and persons seemed largely confident that they could self-sample.

## Supporting information

S1 Appendix Questionnaire data and cross-tabulations. (PDF)
