# Supplementary material for: Diagnostic accuracy and acceptability of molecular diagnosis of COVID-19 on saliva samples relative to nasopharyngeal swabs in tropical hospital and extra-hospital contexts: The COVISAL study
Source: PLoS One. 2021 Sep 13;16(9):e0257169. doi: 10.1371/journal.pone.0257169 (PMC8437265; doi:10.1371/journal.pone.0257169)
Supplement: S1 Appendix — (PDF) [file pone.0257169.s001.pdf]

Supplementary tables: 1-13

Page 2=>Supplementary table 1: Crosstabulation between willingness to repeat a nasopharyngeal test by sex.

Page 2=>Supplementary table 2: Crosstabulation between willingness to repeat a nasopharyngeal test by age group.

Page 3=>Supplementary table 3: Crosstabulation between willingness to repeat a nasopharyngeal test by inclusion site.

Page 3=>Supplementary table 4: Crosstabulation between saliva versus nasopharyngeal test preference by sex.

Page 4=>Supplementary table 5: Crosstabulation between saliva versus nasopharyngeal test preference by age group.

Page 4=>Supplementary table 6: Crosstabulation between willingness to repeat a saliva versus a nasopharyngeal test.

Page 5=>Supplementary table 7: Crosstabulation between motives for refusal to repeat a nasopharyngeal test by sex

Page 5=>Supplementary table 8: Crosstabulation between motives for refusal to repeat a nasopharyngeal test by inclusion site

Page 5=>Supplementary table 9: Crosstabulation between motives for refusal to repeat a nasopharyngeal test by age group

Page 6=>Supplementary table 10: Crosstabulation between motives for refusal to repeat a salivary test by sex

Page 6=>Supplementary table 11: Crosstabulation between motives for refusal to repeat a salivary test by sex

Page 7=>Supplementary table 12: Crosstabulation between feeling capable of preparing a salivary sample alone by sex

Page 8=>Supplementary table 13: Crosstabulation between between feeling capable of preparing a salivary sample alone by age group

Page 9=> Supplementary table 14: Crosstabulation between feeling capable of preparing a salivary sample alone by sampling site

Page 9=>Supplementary table 15: Discordance of result between nasopharyngeal and saliva sample by sampling location

Supplementary table 1: Crosstabulation between willingness to repeat a nasopharyngeal test by sex.

	Sex		
**If your test was negative today, would you be willing to repeat a nasopharyngeal test?**	Males N (%)	Females N (%)	Total N (%)
Refuse	63	120	183
	13.94	20.87	17.82
If necessary	256	274	530
	56.64	47.65	51.61
I would want to do it again	59	60	119
	13.05	10.43	11.59
Doesn't know	30	56	86
	6.64	9.74	8.37
Missing	44	65	109
	9.73	11.30	10.61
Total	452	575	1027
	100.00	100.00	100.00

Chi2 test, P=0.003

Supplementary table 2: Crosstabulation between willingness to repeat a nasopharyngeal test by age group.

	Age group			
**If your test was negative today, would you be willing to repeat a nasopharyngeal test?**	<20 years N (%)	20-40 years N (%)	>40 years N (%)	Total N (%)
Refuse	49	95	39	183
	23.56	21.49	10.34	17.82
If necessary	104	225	201	530
	50.00	50.90	53.32	51.61
I would want to do it again	27	46	46	119
	12.98	10.41	12.20	11.59
Doesn't know	8	36	42	86
	3.85	8.14	11.14	8.37
Missing	20	40	49	109
	9.62	9.05	13.00	10.61
Total	208	442	377	1027
	100.00	100.00	100.00	100.00

Chi2 test: P<0.001

Supplementary table 3: Crosstabulation between willingness to repeat a nasopharyngeal test by inclusion site.

	Inclusion site					
**If your test was negative today, would you be willing to repeat a nasopharyngeal test?**	Cayenne hospital N (%)	Red Cross outreach N (%)	Médecins du Monde N (%)	Maripa soula N (%)	Obstetrics ward N (%)	Total N (%)
Refuse	23	7	41	70	42	183
	19.66	43.75	10.20	17.16	49.41	17.80
If necessary	47	4	232	227	21	531
	40.17	25.00	57.71	55.64	24.71	51.65
I would want to do it again	13	1	80	21	4	119
	11.11	6.25	19.90	5.15	4.71	11.58
Doesn't know	15	2	15	42	12	86
	12.82	12.50	3.73	10.29	14.12	8.37
Missing	19	2	34	48	6	109
	16.24	12.50	8.46	11.76	7.06	10.60
Total	117	16	402	408	85	1028
	100.00	100.00	100.00	100.00	100.00	100.00

Chi 2 test: P<0.001

Supplementary table 4: Crosstabulation between saliva versus nasopharyngeal test preference by sex.

	Sex		
**If the salivary test was validated, which test would you rather get?**	Males N (%)	Females N (%)	Total N (%)
None	1	7	8
	12.50	87.50	100.00
	0.22	1.22	0.78
Nasopharyngeal test	62	52	114
	54.39	45.61	100.00
	13.72	9.04	11.10
Salivary test	278	396	674
	41.25	58.75	100.00
	61.50	68.87	65.63
Does not know	65	60	125
	52.00	48.00	100.00
	14.38	10.43	12.17
Missing	46	60	106
	43.40	56.60	100.00
	10.18	10.43	10.32
Total	452	575	1027
	44.01	55.99	100.00
	100.00	100.00	100.00

Chi2: P=0.009

Supplementary table 5: Crosstabulation between saliva versus nasopharyngeal test preference by age group.

	Age group			
	<20 years	20-40 years N	>40 years	Total
**If the salivary test was validated, which test would you rather get?**	N (%)	(%)	N (%)	N (%)
None	1	3	4	8
	12.50	37.50	50.00	100.00
	0.48	0.68	1.06	0.78
Nasopharyngeal test	22	46	46	114
	19.30	40.35	40.35	100.00
	10.58	10.41	12.20	11.10
Salivary test	153	308	213	674
	22.70	45.70	31.60	100.00
	73.56	69.68	56.50	65.63
Does not know	14	45	66	125
	11.20	36.00	52.80	100.00
	6.73	10.18	17.51	12.17
Missing	18	40	48	106
	16.98	37.74	45.28	100.00
	8.65	9.05	12.73	10.32
Total	208	442	377	1027
	20.25	43.04	36.71	100.00
	100.00	100.00	100.00	100.00

Chi2: P<0.001

Supplementary table 6: Crosstabulation between willingness to repeat a saliva versus a nasopharyngeal test.

**If your test was negative, would you be willing to repeat a salivary test?**	**If your test was negative today would you be willing to repeat a nasopharyngeal test**					
	Refuse	If necessary	I would want to do it again	Doesn't know	Missing	**Total**
Refuse	23	12	11	0	0	**46**
N	*50.00*	*26.09*	*23.91*	*0.00*	*0.00*	**100.00**
(%)	*12.57*	*2.26*	*9.24*	*0.00*	*0.00*	**4.47**
If necessary	89	455	15	23	1	**583**
N	*15.27*	*78.04*	*2.57*	*3.95*	*0.17*	**100.00**
(%)	*48.63*	*85.69*	*12.61*	*26.74*	*0.92*	**56.71**
I would want to do it again	62	55	90	4	1	**212**
N	*29.25*	*25.94*	*42.45*	*1.89*	*0.47*	**100.00**
(%)	*33.88*	*10.36*	*75.63*	*4.65*	*0.92*	**20.62**
Doesn't know	6	7	2	56	2	**73**
N	*8.22*	*9.59*	*2.74*	*76.71*	*2.74*	**100.00**
(%)	*3.28*	*1.32*	*1.68*	*65.12*	*1.83*	**7.10**
Missing	3	2	1	3	105	**114**
N	*2.63*	*1.75*	*0.88*	*2.63*	*92.11*	**100.00**
(%)	*1.64*	*0.38*	*0.84*	*3.49*	*96.33*	**11.09**
**Total**	**183**	**531**	**119**	**86**	**109**	**1028**
N	**17.80**	**51.65**	**11.58**	**8.37**	**10.60**	**100.00**
(%)	**100.00**	**100.00**	**100.00**	**100.00**	**100.00**	**100.00**

Chi2: P<0.001

Supplementary table 7: Crosstabulation between motives for refusal to repeat a nasopharyngeal test by sex.

Why refuse another nasopharyngeal test?	Sex		
	Males<br>N<br>(%)	Females<br>N<br>(%)	Total<br>N<br>(%)
Too much waiting	8	5	13
	61.54	38.46	100.00
Too unpleasant	52	86	138
	37.68	62.32	100.00
Anxiety waiting for result	0	4	4
	0.00	100.00	100.00
Other	4	6	10
	40.00	60.00	100.00
Total	64	101	165
	38.79	61.21	100.00

Chi2: P=0.14

Supplementary table 8: Crosstabulation between motives for refusal to repeat a nasopharyngeal test by inclusion site

	Inclusion site					
**Why refuse another nasopharyngeal test?**	Cayenne hospital<br>N<br>(%)	Red Cross outreach<br>N<br>(%)	Médecins du Monde<br>N<br>(%)	Maripa soula<br>N<br>(%)	Obstetrics ward<br>N<br>(%)	Total<br>N<br>(%)
Too much waiting	2	1	5	5	0	13
	15.38	7.69	38.46	38.46	0.00	100.00
Too unpleasant	21	3	23	57	34	138
	15.22	2.17	16.67	41.30	24.64	100.00
Anxiety waiting for result	1	0	0	2	1	4
	25.00	0.00	0.00	50.00	25.00	100.00
Other	6	0	3	1	0	10
	60.00	0.00	30.00	10.00	0.00	100.00
Total	30	4	31	65	35	165
	18.18	2.42	18.79	39.39	21.21	100.00

Chi2: P=0.01

Supplementary table 9: Crosstabulation between motives for refusal to repeat a nasopharyngeal test by age group

	Age group			
**Why refuse another nasopharyngeal test?**	<20 years<br>N<br>(%)	20-40 years<br>N<br>(%)	>40 years<br>N<br>(%)	Total<br>N<br>(%)
Too much waiting	4	6	3	13
	30.77	46.15	23.08	100.00
Too unpleasant	35	77	26	138
	25.36	55.80	18.84	100.00
Anxiety waiting for result	0	4	0	4
	0.00	100.00	0.00	100.00
Other	0	1	9	10
	0.00	10.00	90.00	100.00
Total	39	88	38	165
	23.64	53.33	23.03	100.00

Chi 2: P<0.001

Supplementary table 10: Crosstabulation between motives for refusal to repeat a salivary test by sex

**Why would you refuse another salivary test?**	**Sex**		
	Males<br>N<br>(%)	Females<br>N<br>(%)	Total<br>N<br>(%)
Too much waiting	8<br>47.06	9<br>52.94	17<br>100.00
Too unpleasant	12<br>38.71	19<br>61.29	31<br>100.00
Anxiety waiting for result	0<br>0.00	2<br>100.00	2<br>100.00
Other	7<br>50.00	7<br>50.00	14<br>100.00
Total	27<br>42.19	37<br>57.81	64<br>100.00

Chi 2: P=0.5

Supplementary table 11: Crosstabulation between motives for refusal to repeat a salivary test by age group

**Why would you refuse another salivary test?**	Age group			
	<20 years<br>N<br>(%)	20-40 years<br>N<br>(%)	>40 years<br>N<br>(%)	Total<br>N<br>(%)
Too much waiting	3<br>17.65	10<br>58.82	4<br>23.53	17<br>100.00
Too unpleasant	6<br>19.35	15<br>48.39	10<br>32.26	31<br>100.00
Anxiety waiting for result	0<br>0.00	1<br>50.00	1<br>50.00	2<br>100.00
Other	0<br>0.00	5<br>35.71	9<br>64.29	14<br>100.00
Total	9<br>14.06	31<br>48.44	24<br>37.50	64<br>100.00

Chi2: P=0.25

Supplementary table 12: Crosstabulation between feeling capable of preparing a salivary sample alone by sex

	Sex		
**Would you feel capable of giving a saliva sample test on your own?**	Males N (%)	Females N (%)	Total N (%)
No	27	28	55
	49.09	50.91	100.00
	5.97	4.87	5.36
Yes, very easily	272	338	610
	44.59	55.41	100.00
	60.18	58.78	59.40
Yes, but uneasy about it	37	43	80
	46.25	53.75	100.00
	8.19	7.48	7.79
Yes, with a nurse's help	16	17	33
	48.48	51.52	100.00
	3.54	2.96	3.21
Maybe	14	33	47
	29.79	70.21	100.00
	3.10	5.74	4.58
Doesn't know	40	46	86
	46.51	53.49	100.00
	8.85	8.00	8.37
Missing	46	70	116
	39.66	60.34	100.00
	10.18	12.17	11.30
Total	452	575	1027
	44.01	55.99	100.00
	100.00	100.00	100.00

Chi2: P=0.41

Supplementary table 13: Crosstabulation between feeling capable of preparing a salivary sample alone by age group

	Age group			
**Would you feel capable of producing a saliva sample on your own?**	<20 years N (%)	20-40 years N (%)	>40 years N (%)	Total N (%)
No	14	23	18	55
	25.45	41.82	32.73	100.00
	6.73	5.20	4.77	5.36
Yes, very easily	124	274	212	610
	20.33	44.92	34.75	100.00
	59.62	61.99	56.23	59.40
Yes, but uneasy about it	29	27	24	80
	36.25	33.75	30.00	100.00
	13.94	6.11	6.37	7.79
Yes, with a nurse's help	3	12	18	33
	9.09	36.36	54.55	100.00
	1.44	2.71	4.77	3.21
Maybe	8	23	16	47
	17.02	48.94	34.04	100.00
	3.85	5.20	4.24	4.58
Doesn't know	8	38	40	86
	9.30	44.19	46.51	100.00
	3.85	8.60	10.61	8.37
Missing	22	45	49	116
	18.97	38.79	42.24	100.00
	10.58	10.18	13.00	11.30
Total	208	442	377	1027
	20.25	43.04	36.71	100.00
	100.00	100.00	100.00	100.00

Chi2: P=0.003

Supplementary table 14: Crosstabulation between feeling capable of preparing a salivary sample alone by sampling site

	Inclusion site					
**Would you feel capable of producing a saliva sample on your own?**	Cayenne hospital N (%)	Red Cross outreach N (%)	Médecins du Monde N (%)	Maripa soula N (%)	Obstetrics ward N (%)	Total N (%)
No	4	1	19	27	4	55
	7.27	1.82	34.55	49.09	7.27	100.00
	3.42	6.25	4.73	6.62	4.71	5.35
Yes, very easily	65	9	273	210	54	611
	10.64	1.47	44.68	34.37	8.84	100.00
	55.56	56.25	67.91	51.47	63.53	59.44
Yes, but uneasy about it	9	0	24	46	1	80
	11.25	0.00	30.00	57.50	1.25	100.00
	7.69	0.00	5.97	11.27	1.18	7.78
Yes, with a nurse's help	7	1	13	9	3	33
	21.21	3.03	39.39	27.27	9.09	100.00
	5.98	6.25	3.23	2.21	3.53	3.21
Maybe	3	3	21	15	5	47
	6.38	6.38	44.68	31.91	10.64	100.00
	2.56	18.75	5.22	3.68	5.88	4.57
Doesn't know	7	1	15	52	11	86
	8.14	1.16	17.44	60.47	12.79	100.00
	5.98	6.25	3.73	12.75	12.94	8.37
Missing	22	1	37	49	7	116
	18.97	0.86	31.90	42.24	6.03	100.00
	18.80	6.25	9.20	12.01	8.24	11.28
Total	117	16	402	408	85	1028
	11.38	1.56	39.11	39.69	8.27	100.00
	100.00	100.00	100.00	100.00	100.00	100.00

P<0.001

Supplementary table 15: Discordance of result between nasopharyngeal and saliva sample by sampling location

Discordance	Adjusted OR	[95% Confidence Interval]	p-value
Reference Cayenne	-	-	-
Doctors of the World	6.6	1.6-26.6	0.008
Maripasoula	1.1	0.3-3.8	0.7
Obstetrics	-	-	-
Asymptomatic	9.9	5.1-19.2	0.007
